# What interventions are required to reduce road traffic injuries in Africa? A scoping review of the literature

**DOI:** 10.1371/journal.pone.0208195

**Published:** 2018-11-30

**Authors:** Emmanuel Bonnet, Lucie Lechat, Valéry Ridde

**Affiliations:** 1 Institut de Recherche pour le Développement, UMI Résiliences, Bondy, France; 2 AGIR, Ouagadougou, Burkina Faso; 3 Institut de Recherche pour le Développement, UMR CEPED, Université Paris Descartes, Paris, France; Western University, CANADA

## Abstract

Road traffic accidents are the major cause of mortality among people aged 15–29 years in Africa. World Health Organisation (WHO) and the World Bank launched a Decade of Action for Road Safety in 2011 with the goal of halving the number of injuries and deaths on the roads. No progress has been reported in Low and Middle Income Countries (LMICs) and the number of deaths remains very high. To reach the target set, there is a need for interventions in several areas. This scoping review proposes to produce a synthesis by identifying the kinds of interventions and outcomes which have been carried out on the African continent. Using the scoping studies method, 23 articles were selected and analysed. The study shows that interventions were developed in four fields: road safety policy, health education, safety equipment and data collection. It shows also that there were records of interventions in only twelve countries, mostly in Eastern and Southern Africa. The main conclusion of this study reveals both a lack of road safety interventions and shortcomings in the assessment of those performed and selected for our study.

## Introduction

Road Traffic Injuries (RTIs) are the eighth cause of death in the world and there has been a 46% increase since the 1990s [1]. Recent WHO analyses estimate that RTIs could become the fifth cause of death in the world by 2030 with high levels of inequality in situations, between, and within, LMICs (Low and Middle Income Countries) [2]. Added to this burden are the millions of people suffering long-term from their injuries or disabilities [3].

The highest number of deaths occurs on the African continent, i.e. a rate of 26.6 deaths per 100 000 inhabitants [2]. A recent study found that the rate could be closer to 65 deaths per 100 000 inhabitants [4]. Furthermore, young Africans are the most likely victims with road traffic accidents being the first cause of mortality among 15–29-year-olds [2]. Economically, the average annual socio-economic cost of road traffic crashes represents 1% of Gross National Product (GNP) in low-income countries[5]. With the rapid rise of motorisation in Africa, action must be taken to reduce accidents and protect populations.

The Decade of Action for Road Safety 2011–2020, developed by WHO and the World Bank, provides an overall framework for road safety activities [6] based on five pillars, which are: better management of road safety (most notably legislation), better road safety (e.g. improving safety standards), better safety of vehicles, better safety for road users (e.g. wearing helmets), and improvement in trauma and recovery management (training staff in proper practices). If a fair number of them are still ongoing, recent interventions have demonstrated their potential effectiveness in certain LMICs. In Cambodia, for example, encouragement of helmet use has been effective with a reduction in the number of deaths and serious head injuries [7]. The same activities have not been implemented in every country and, to our knowledge, few interventions have been developed on the African continent to reduce this burden [8].

Although the Decade of Action for Road Safety has been underway for 8 years, the number of deaths has remained high and there has been no significant improvement. Half-way through the decade, mortality rates have stagnated worldwide. The number of fatalities is still high in LMICs, and is increasing each year [2]. In order to keep up the activities of the Decade of Action for Road Safety, sustainable development goals included a powerful ambition in their 3.6 target: to reduce the number of road traffic deaths and injuries by 50% by 2020 [9]. Furthermore, Sustainable Development Goals (SDG) 3.6 (number of global deaths and injuries from road traffic accidents) and 11.2 (improving road safety) have one of the most “indivisible” interactions among all the SDGs according to a recent report of experts [10].

To reach this 3.6 target, and looking beyond interventions on infrastructures, the effectiveness of which is understood, interventions must be conducted in the fields of awareness-raising, provision of rescue services and also public policies and regulations which contribute to the reduction in accidents and their impact on health. Therefore, the purpose of this article is to produce a state of knowledge on the interventions that have been implemented on the African continent.

The objective of this article is to provide a state of knowledge on interventions to reduce road accidents and injuries in Africa. This paper describes the interventions, their results and evaluation methods. Our synthesis complements two recently published systematic reviews on the topic of Road Traffic Injuries in Africa. Staton’s review [11], published in 2016, focuses on articles which present interventions linked to prevention, with an assessment of the impact on the number of accidents, accidents with injuries and deaths. The systematic review targets all LMICs. The findings show that of the 18 articles selected, only four were based in sub-Saharan Africa. Interventions were concerned with road improvement, speed control, legislation and an increase in police enforcement. Vissoci’s systematic review [12], published in 2017, analysed the proportion of injured persons per road accident admitted to hospitals in sub-Saharan African countries. It identified 13 African countries. The findings revealed that almost 30% of patients admitted with injuries had road traffic traumas. The article also mentioned that the number of studies on the subject remained low, despite WHO and the Decade of Action for Road Safety having alerted them to the need to produce fresh knowledge. Our scoping review draws up a list of all the interventions performed on the continent with far more open selection criteria in order to have more wide-ranging information about the interventions. Indeed, these two systematic reviews did not present the details of the interventions, their content, implementation and context. Our approach aims to go into the details of complex interventions as recommended in intervention research [12] in order to identify methods and actions that have been effective. However, these two recent publications illustrate not only the urgency of the RTI issue in Africa but also underline the actions performed and the efforts that need pursuing.

## Materials and methods

The principal goal of this review is to take stock of the knowledge of interventions aiming to reduce the number of accidents and injuries. By intervention, we mean “a coherent, organised and structured set of objectives, activities, means and people who conduct it, implemented in order to transform a problematic situation.” [13]. The search for references was completed in December 2017.

The methodology of research employed is based on the one developed by Arskey and O’Malley [14]. It comprises five principal stages.

### 1-Definition of the research question and eligibility criteria

The research question endeavours to identify, take stock of, describe and analyse all the interventions that aim to reduce road traffic accidents and injuries in Africa. We have chosen to include all publications known since the 1950s in order to identify all types of interventions that have been carried out in Africa so as to enumerate them and highlight successful experiences. To address this question, the two principal eligibility criteria for selecting the papers for this scoping review concern i) the geographic area (countries on the African continent), ii) the fields of interventions (prevention, support, regulations). Implicitly, the selected interventions do not target specific ages, specific methods or geographical environments. The selected interventions must be detailed enough to complete the Tidier grid [15]. All primary research articles have been included considering the period 1950–2018.

### 2-Sources of bibliographic data consulted

Several databases were used [16]: Sciencedirect and Pubmed, EMBASE, CINAHL, MEDNAR, CENTRALE PsycINFO and la Banque de données en santé publique (public health database).

Database queries were adapted to each database, in accordance with the appropriate request vocabulary. For certain bases, the performance of query criteria (Booleans) is more important. This is the case, for example, for MeSH terms in the MEDLINE and CENTRAL databases (S1 Appendix).

Additionally, a search into grey literature was conducted as well as a manual search from article references. The OpenGrey, National Institute for Health and Clinical Excellence (NICE), National Guideline Clearinghouse, Social Care Online and The Grey Literature Report databases were therefore used to identify the literature. Lastly, a final analysis was conducted in Google Scholar to identify papers not listed in the aforementioned databases.

### 3-Search strategy

The search strategy focused on references in French and English as described below (S1 Appendix):

("accident traffic" OR "road traffic injury" OR "Road safety" OR "Road crash" OR "road accident")

                             **AND**

(“accident prevention” OR “accessibility of health services” OR “access to health care” OR “accessibility, health services” OR “health intervention” OR “development, policy” OR “efficiency, program” OR “effectiveness research, comparative” OR “effectiveness” OR “efficiency”)

                             **AND**

("africa" OR "west africa" OR "Burundi" OR "republic of Burundi" OR "Angola" OR "Algeria" OR "Benin" OR "republic of Benin" OR "Comoros" OR "iles Comores" OR "Cameroon" OR "republic of Cameron" OR "united republic of Cameroon" OR "Egypt" OR "arab republic of Egypt" OR "Burkina Faso" OR "burkina faso" OR "Djibouti" OR "republic of Djibouti" OR "Central African Republic" OR "Libya" OR "Cape Verde" OR "republic of cape verde" OR "Eritrea" OR "Chad" OR "Morocco" OR "Cote d’Ivoire" OR "ivory coast" OR "Ethiopia" OR "federal democratic republic of Ethiopia" OR "Congo" OR "congo Brazzaville" OR "congo Kinshasa" OR "Sudan" OR "republic of the sudan" OR "Gambia" OR "republic of the gambia" OR "Kenya" OR "republic of Kenya" OR "Democratic Republic of the Congo" OR "Tunisia" OR "Ghana" OR "republic of Ghana" OR "Madagascar" OR "Guinea" OR "Malawi" OR "republic of Malawi" OR "Equatorial Guinea" OR "republic of equatorial guinea" OR "Guinea-Bissau" OR "republic of guinea Bissau" OR "Mauritius" OR "Gabon" OR "Liberia" OR "republic of Liberia" OR "Mozambique" OR "republic of Mozambique" OR "Sao Tome and Principe" OR "Mali" OR "Reunion" OR "Botswana" OR "Mauritania" OR "Rwanda" OR "republic of Rwanda" OR "Lesotho" OR "kingdom of Lesotho" OR "Niger" OR "republic of niger" OR "Seychelles" OR "Namibia" OR "republic of Namibia" OR "Nigeria" OR "federal republic of Nigeria" OR "Somalia" OR "St Helena" OR "saint Helena" OR "Uganda" OR "republic of Uganda" OR "Swaziland" OR "Senegal" OR "republic of Senegal" OR "United Republic of Tanzania" OR "South Africa" OR "Sierra Leone" OR "republic of sierra leone" OR "Zambia" OR "republic of Zambia" OR "Togo" OR "Zimbabwe" OR "republic of Zimbabwe" OR "zimbabwe rhodesia")

To optimise the query, the names of all African countries and their regional names were added to the search strategy as well as the term “africa” so as to maximise the selections coming from titles and keywords. The search strategy was also given in French. We sought the expertise of a librarian for the definition of the search strategy.

### 4-Selection of studies

The selection of studies was done in four stages representing four triage phases of the references selected: general identification, screening, eligibility and lastly selection of the articles included.

**Fig 1:**
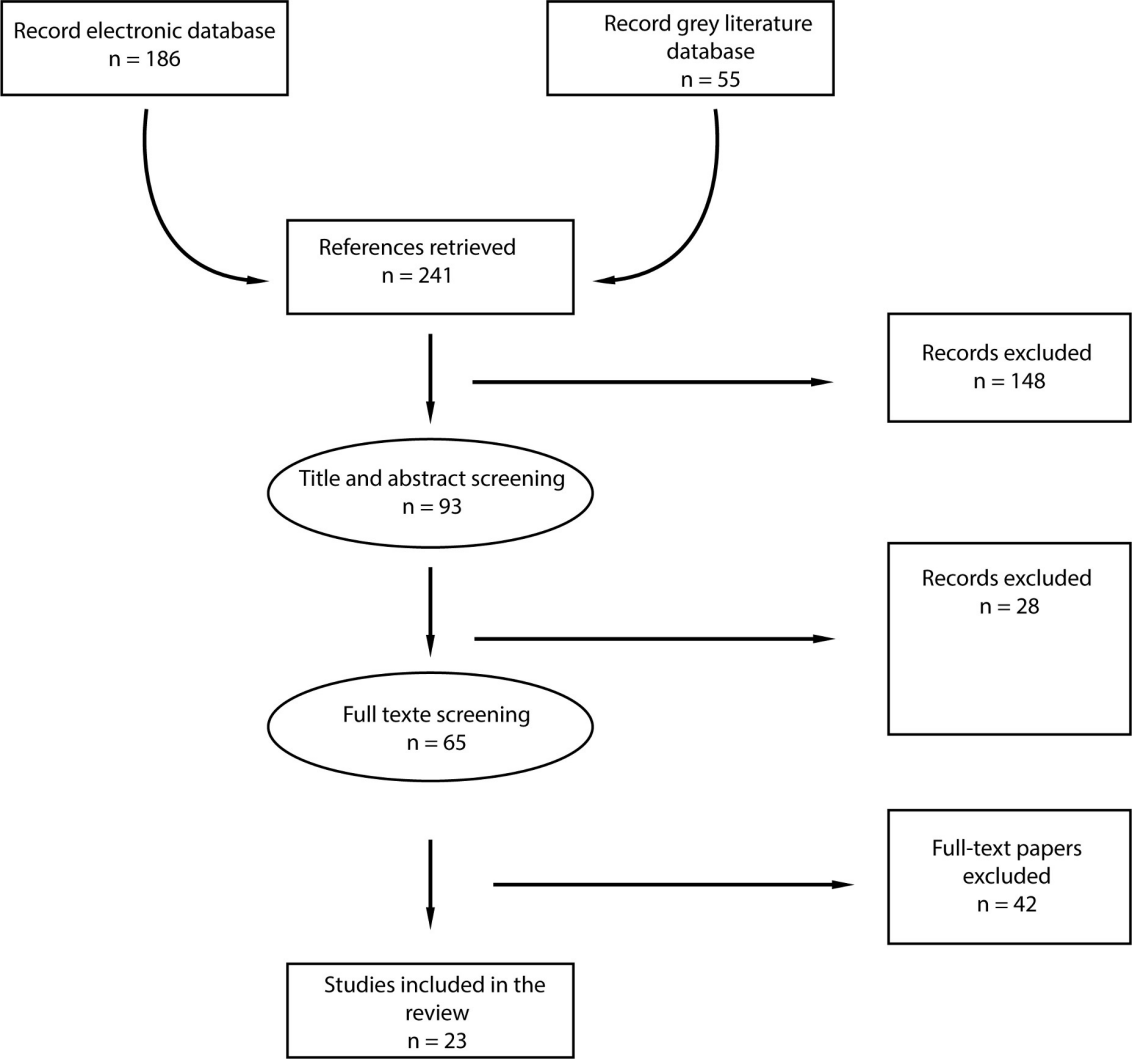
“PRISMA” flowchart.

The first two authors completed the selection of the publications. They assessed the titles and abstracts independently. The selection was then compared to retain the list of publications included. Any rare cases of disagreement (n = 8) were dealt with by consensus. The reasons for exclusion of the articles were mainly associated with a geographical area of intervention outside Africa; the description of legislation or public policies not implemented in the field; and theoretical descriptions of interventions that could be carried out to reduce trauma but not applied. Most of the excluded articles included in their titles or summaries a reference to interventions, but they were not carried out. Details of the selection can be found in the presentation of the PRISMA flowchart (Fig 1) and PRISMA Checklist (S3 Appendix).

### 5-Analysis strategy

The analysis grid used was taken from the template for intervention description and replication (TIDieR) checklist (Appendix 2) [11]. It is based on twelve elements used to describe an intervention: Name, Why, What (material), What (process), Who delivered, How, Where, When, Adaptation (tailoring), Modifications, Assessment—planned and real. However, we completed the TIDieR with contextual elements in order to gain a better understanding of an intervention [17]. Therefore, seven contextual aspects were added to the analysis grid: population, settings, the political, economic, socio-cultural, historical environment and health.

## Results

The search allowed us to identify 241 articles. They were compiled in the Zotero bibliographic software tool which made it possible to sort through the references and remove duplicates. Once the four triage phases had been completed, 23 articles had been retained. Grey literature documents (n = 55) were not included in the final selection as they did not deal exclusively with detailed interventions, but with one-off actions included in an overall approach to road safety. Fig 1 summarises the final selection.

Table 1 presents all the selected articles, their types of intervention as well as the criteria for this scoping review.

**Table 1:** Table of selected articles.

Intervention	Author(s)	Year	Country	Objectives	What (material or procedure)	When	Effectiveness control	Effectiveness (yes, no, partially)	Sustainability	Scaled up
**Health education**	Jones Blantari et al.	2005	Ghana	Reduce the RTIs caused by overspeeding and drink-driving	Spot radio	Once time by week during 9 month between 2002 and 2003	No	No informations	No	No
	Lipinge et al.	2014	Namibia	Use mass media to make preventive on RTIs	Mass media No details	No details	Meta-analyzes	No informations	No	No
	Bili Priscilla	2015	Zambia	Reduce the number of pedestrian accident among children	awarness campaing TV, radio, Print media (" …pamphlets, brochures and posters to increase public awareness. It also uses Billboards), drama and musical concert, road safety week, community publicity campaigns at traditional ceremony, road safety school club, day of remembrance	No details	No	Partially	No	No
	Petroze Robin T. and al	2014	Rwanda	Improve the cover of first care for injuries	educational short-course curricula Advanced Trauma Life Support (ATLS) or Trauma’s Trauma Team Training (TTT)	October–November, 2011	Retrospective cohort study	Yes	Yes	No
	O. E. Johnson and E. T. Owoaje	2012	Nigeria	Implement Health Education on the Riding Habits of Commercial Motorcyclists	Training session	3 month	RCT	Yes	No	No
	Johnson O. E., and A.M. Adebayo	2011	Nigeria	impact on the knowledge and the conformity of the signs of road safety at the commercial motorcyclists	Visuals aids	september 2008	RCT	Partially	Yes	No
	James Habyarimana and William Jack	2011	Kenya	Put stickers in vehicles to incite the passengers to speak and to check if drivers has no adapted driving	Visuals aids with Stickers	between january 2006 and may 2009	RCT	Yes	No	No
	James Habyarimana and William Jack	2015	Kenya	Put stickers in vehicles to incite the passengers to speak and to check if drivers has no adapted driving	Visuals aids with Stickers and media campaign	From 2011 to 2013 The campaign was activated on a weekly basis on five occasions over the course of the first 6 mo of 2012.	RCT	Yes	No	No
**road safety policy**	Teferi Abegaz, et al.	2014	Ethiopia	improved road safety policy	New road safety laws	After 2007	Interrupted time series design	Partially	No	No
	A. P. ROSE-INNES and C. J. G. LE ROUX	1974	South Africa	Change of behaviors with over-speeding and Influence of Road Speed Restrictions on the Incidence and Severity of Head Injuries	Filling stations closed between 18h hand 06h the next morning and at weekend + speed limits of 60 km/h in urban areas (initially 50 km/h for 2 months), and 80 km/h outside these areas Media campaigns have also been implemented	After november 1973	Retrospective cohort study	Yes	No	No
**Policy and program safety** **(global/mixte)**	Peter Anderson, et al.	2009	LMICs	reduce the avoidable harm caused by alcohol	1) Information and education 2) health-sector response (Brief advice for individdual no dependant, specialised treatment) 3) Community programmes (media advocacy, Community interventions, Workplace policies) 4) Drink-driving (Introduction and/or reduction of alcohol concentration in the blood, Sobriety checkpoints and unrestrictive (random) breath testing, Restrictions on young or inexperienced drivers (eg, lower concentrations of alcohol in blood for novice drivers), Mandatory treatment, Alcohol locks, Designated driver and safe-ride programmes) 5) addressing the availability of alcohol (Government monopolies, Minimum purchase age, Outlet density, Days and hours of sale) 6) addressing the marketing of alcoholic beverages (Volume of advertising, Self-regulation of alcohol marketing, 7) pricing policies (Alcohol taxes) 8) harm reduction (Training of bar staff, responsible serving practices, security staff in bars, and safety-oriented design of the premise) 9) reducing the public health eff ect of illegally and informally produced alcohol (Informal and surrogate alcohols, Strict tax labelling)	No details	Meta-analyses	Yes	No	No
	Terje Assum	1998	Republic of Benin	Reduce RTAs	1) Create a database on RTAs; 2) Set up an awareness and education program; 3) improve vehicle technical control centers. No more details	1995	Meta-analyzes	No	No	No
			Ivory cost	Reduce RTAs	1) Improve vehicle technical control; 2) Equipping buses with speed limiters; 3) Improve vehicle controls by police and gendarmerie; 4) Create a professional driver's license (there is currently only a general driving license); 5) Provide equipment and human resources to the Road Safety Authority—train drivers and professional drivers; 6) Amend the rules governing driving schools; 7) restructure the Road Safety Authority; 8) Establish 3 itinerant committees to "control controllers"; 9) Launch national and regional awareness campaigns on road safety; 10) Treat blackheads.	> 1998	Meta analyzes	No	No	No
			Kenya	Reduce RTAs	24 measures: enforcement, establishment of an accident investigation committee, driver training program, vehicle technical inspection, road planning and maintenance program, First-aid courses, information and education campaigns, and research on road safety.	1980	Meta-analyzes	Yes	No	No
			Tanzania	Reduce RTAs	1) Establish a mechanism capable of integrating problems through a multisectoral approach, through short- and long-term road safety plans; 2) reducing the risk of accidents and minimizing their consequences; 3) Extend the life of the road network by ensuring effective vehicle and axle load control.	1996	Meta-analyzes	No	No	No
			Zimbabwe	Reduce the RTAs rate by 15%	1) relieving medical services; 2) replace vehicles and equipment acquired in 1986; 3) present the road safety action plan to 25,000 teachers; 4) outline an upcoming program for the years 2001–2005 on the basis of the experience acquired or renew the existing program if it has not been completed; 5) submitting to a training program some 10 000 pupils living in rural areas.	5 years—no details	Meta-analyzes	No	No	No
	A Aeron-Thomas, et al.	2002	Ethiopia, Ghana, South Africa, Zambia	Reduce RTAs	Four thems : - organisation of road safety - road safety plans - funding of road safety - private sector participation	No details	Meta-analyzes	Partialy	No	No
	D Bishai, B Asiimwe, S Abbas, A A Hyder, W Bazeyo	2008	Kampala, Uganda	Reduce RTAs	Foot patrols with radar on main road	since 2004	RCT	Yes	Don't know	Don't know
**Safety equipment**	"Steven A. Sumner, Anthony J. Pallangyo et al.	2014	Tanzania	improve their abilty to be seen by other vehicules	Conspicuity equipment and reflect vest	3 month—2013	RCT	Partialy	No	No
	Liu BC, Ivers R et al.	2008	Around the world	reducing mortality and head, face and neck injury following motorcycle crashes	wearing helmet	No detail	Meta-analyses	Yes	No	No
	Karen Zimmerman, et al.	2015	Tanzania	Reducing road safety-related mortality and trauma by implementing a road safety program for a rural community	safety equipments (Reflective vests and helmets, back vest, reflective bag, reflective stickers, calendar) + short courses	Trainig for motocyclists was one week No more information concerning other training	No	No	No	No
	Milton Mutto, et al.	2002	Uganda	Reduce pedestrian accidents with overpass	Overpass	1999	No	Partialy	No	No
	N Nadesan-Reddy, S Knight	2013	South Africa	Introduce traffic calming measures to decrease the speeding	Speed hump In parallel with the implementation of speed bumpers, there were several other safety measures: speed limits and road design, the introduction of road safety education into the curriculum of primary school and The employment of 'school point supervisors' to a few schools to help at studded crossing points.	From 2001	Interrupted time series design	Yes	No	No
	Obionu, C.N., Asogwa, S.E.	1985	Nigeria	Reduce the pedestrians accidents To put pedestrian safety measures (overpass) to protect the pedestrian near a school	Overpass near school	No detail	Retrospective cohort study	Partialy	No	No
	N.A. Ebot Eno Akpa, M.J. (Thinus) Booysen, M. Sinclair,	2015	South Africa	Introduce countermeasures to decrease the speeding	rumble strips, speed humps, and instantaneous speed cameras strategically deployed on roads	Since 2011	Retrospective cohort study	partially	No	No
**Data collection**	Adebayo Peter Idowu,et al.	2015	Nigeria	monitoring system can be used by the security guards road to react quickly to the road accidents (improve the answer to the first aid), investigate and record these events, allow the other actors of the road traffic to consult files and to make decisions to reduce the road accidents	a web-based road traffic accident monitoring system to replace the existing paper	Just theory, not implemented	No	No	No	No
	Gbadamosi Kolawole	2015	Nigeria	Analysis through GIS has the advantage of being a decision-making aid and implementing targeted measures to reduce RTAs in Nigeria. It's to give elements of help to the decision It's an indirect measure	GIS	Analyse RTA data between 1990 and 2012	No	Partialy	No	No

### Geographic distribution

**Fig 2:**
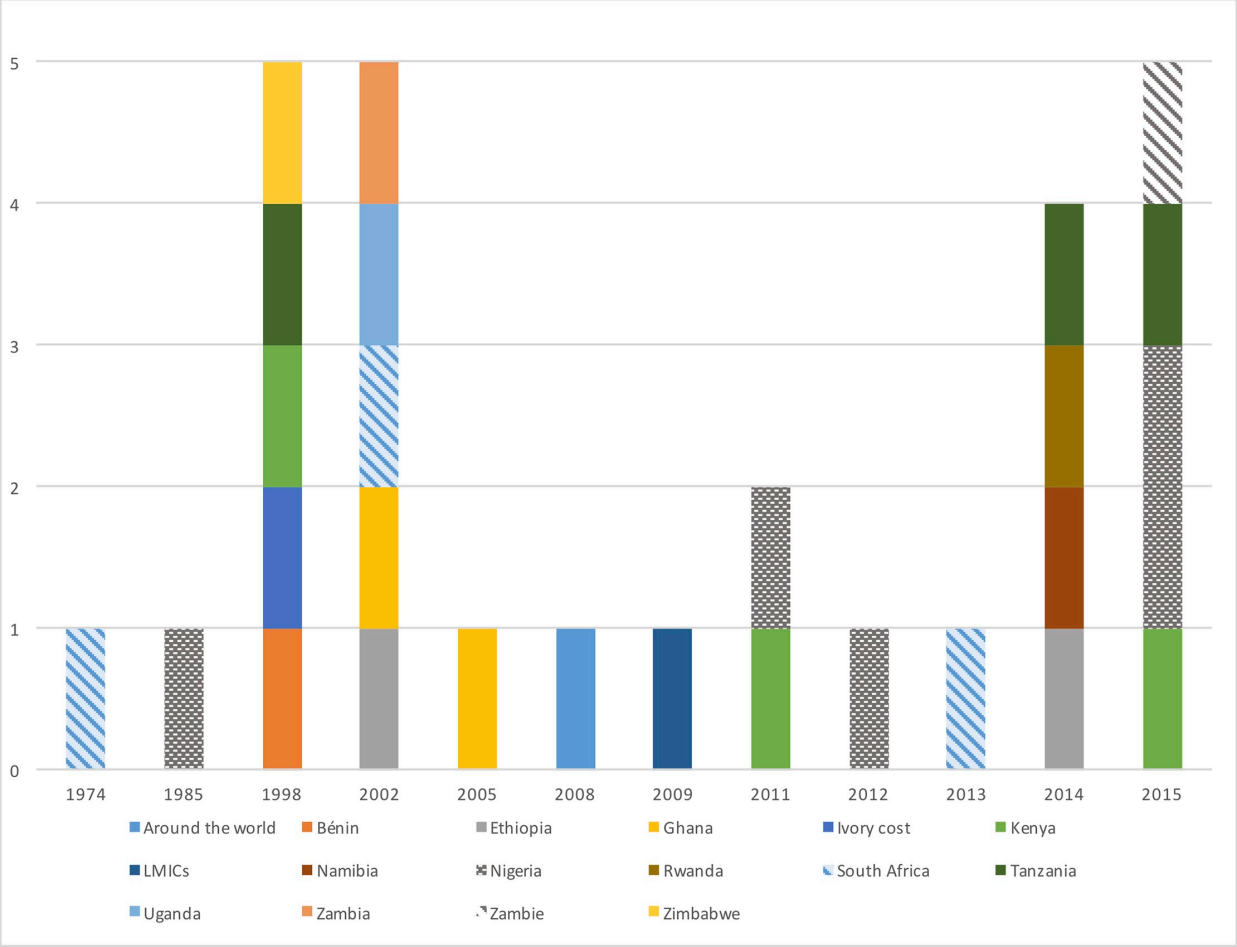
Distribution of documents included by year and by country.

Most of the publications focused on the last 20 years, with an increase since 2014 (Fig 2 2). Nigeria (n = 5), Tanzania (n = 3), Kenya (n = 3) and South Africa (n = 3) had been studied the most, representing almost 61% of all the articles. These countries have long been studied but an increase in the number of articles has been seen, since 2010, in Kenya, Tanzania, and Nigeria. With regard to geographic areas, southern and eastern Africa were the most frequently studied countries (60%). Central and northern Africa were not represented in any articles proposing an intervention. Lastly, West Africa received uneven treatment, with the main focus on Ghana and Nigeria.

**Fig 3:**
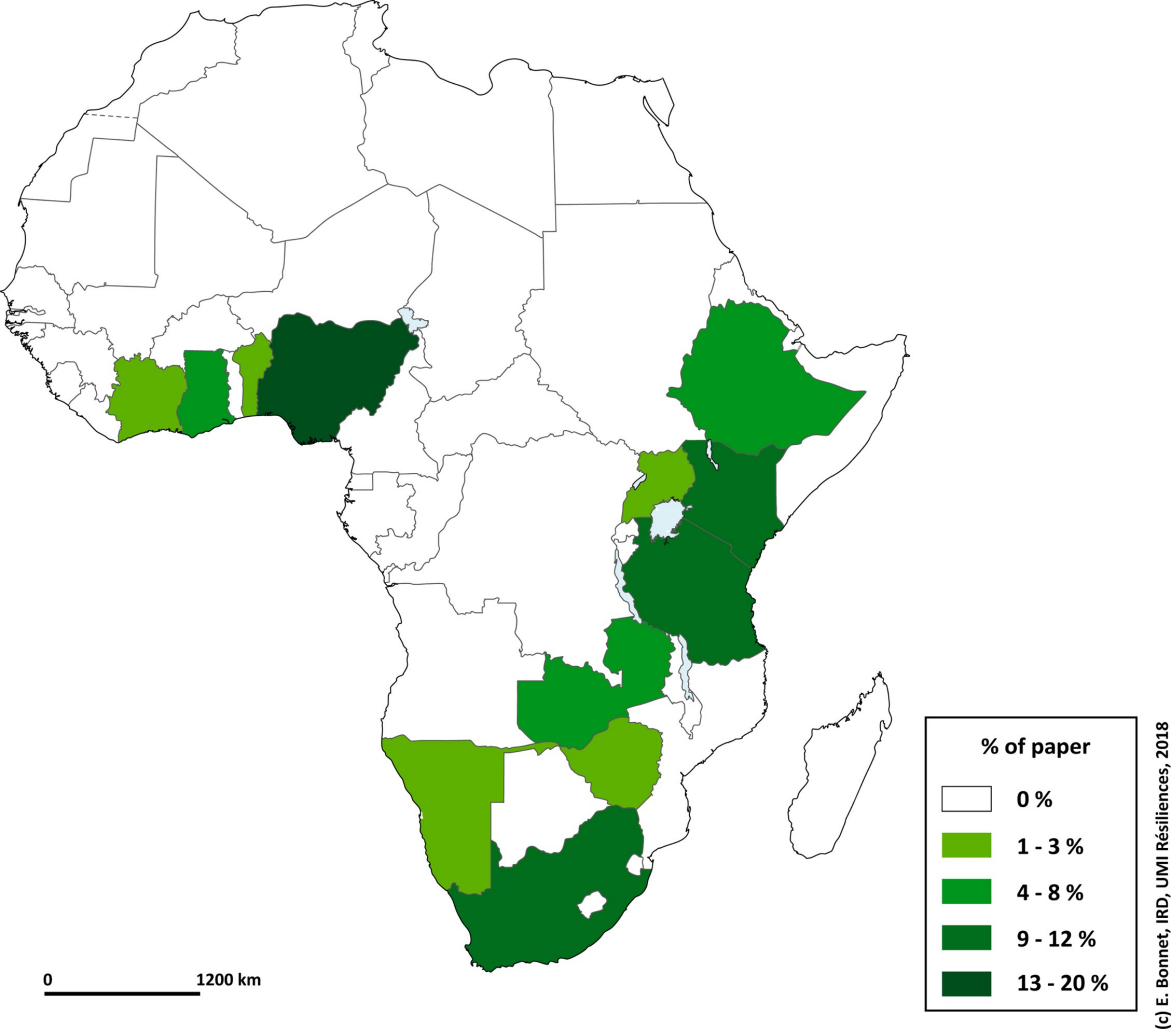
Location of areas where interventions were implemented.

Mapping the distribution (Fig 3) of publications revealed two principal zones covered by published interventions. One zone is in the Gulf of Guinea, corresponding to one of the most important road transport corridors [18] in West Africa: the Abidjan–Lagos corridor. The most frequently covered area corresponds to countries in East-Central Africa as far as South Africa, countries where there has been greater economic development and where levels of motorisation are the highest on the continent. [19].

### Description of interventions

**Fig 4:**
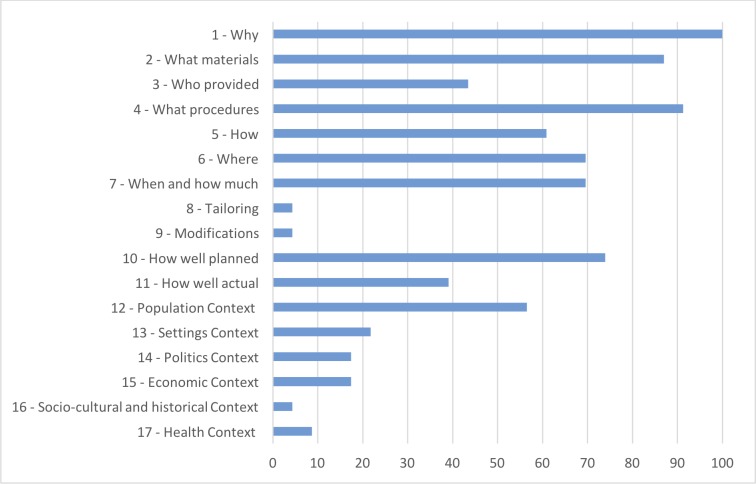
Tidier checklist.

We analysed the interventions using an analysis grid with 17 criteria according to the Tidier grid. Fig 4 shows how the interventions were described in each selected article in relation to the size of our analysis grid. If the general description of the intervention was broadly included in the publications, it was noted that some important information for a potential replication was missing. Indeed, only one article presented the adaptation and modifications of the intervention implemented. The complete grid is available in S2 Appendix for access to all the results of the control list.

#### Fields of interventions

**Fig 5:**
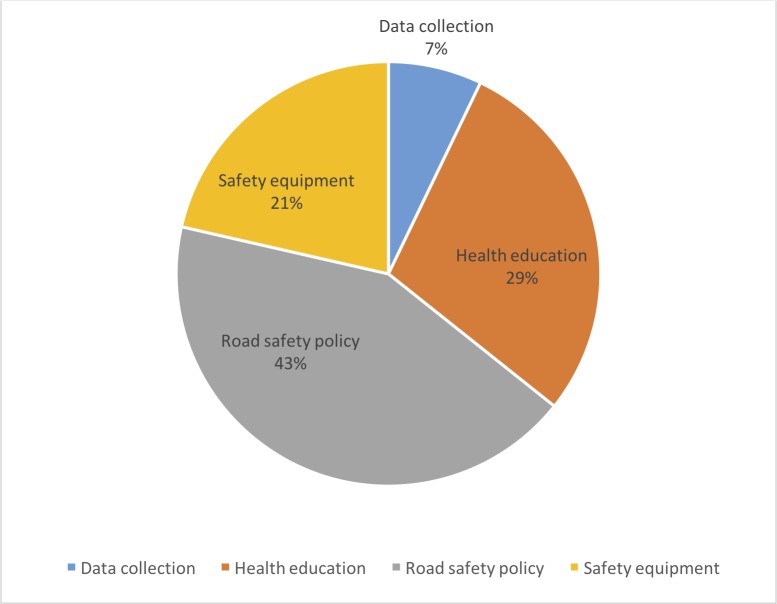
Distribution of the fields of intervention.

Analysis of the publications identified four principal fields of intervention (Fig 5). The most commonly addressed was health education (n = 8) but has only recently been implemented (since 2005). The second field was safety equipment (n = 7), the oldest and most frequently applied over the last 15 years. The third field was public policies undertaken (n = 6). Lastly, data collection (n = 2), which is very recent (2014), coupled with innovative techniques, represented the final field, which was addressed only in Kenya. Data collection is regarded as an intervention as it makes it possible to integrate new technologies into monitoring and assessing road traffic accidents and injuries and therefore targets which actions to undertake. Therefore there were no publications addressing interventions across the entire spectrum of the pillars [6] of road traffic accidents and their consequences.

### Goals and types of intervention

Five types of intervention were distinguished and were unevenly distributed according to their fields. The media, for example, covered five of the eight interventions in the field of health education. Other interventions, whose overall objective was a reduction in accidents, were actually a combination of several types of intervention. In Côte d’Ivoire, a public policy proposed 10 types of intervention [20] at the same time, for example, providing equipment to limit bus speeds, supporting national prevention campaigns and creating new laws for driving licences. These types of intervention provided information about the implementation and the panel explored in an attempt to reduce the burden of road traffic accidents.

#### 1 –Media

Of the 23 articles, eight focused on the assessment of media activities in road safety. Three articles pointed to campaigns implemented in the development of vast road safety programmes in Kenya as early as 1980 [20], Namibia [21] and more generally in the LMICs [22]. However, these three references did not provide details on the type of communication channel, content of messages and their frequency.

The goal of communication campaigns is to warn and raise awareness in connection with a given problem. Some campaigns provided information on dangerous driving [22–24] or targeted a category of road traffic accident victims, like children [25] for example.

In Ghana [23], television spots were broadcast in English and Akan once a month for nine months between 2002 and 2003. The target was commercial drivers, seen as dangerous drivers (speeding and under the influence of alcohol). Assessment of this intervention highlighted the need to turn to other channels of dissemination (radio, leaflets, etc.) to reach more people, not all of whom had access to television. Although the messages were well received and appeared simple, the use of other languages was reported as necessary. It was also mentioned that parallel activities should be implemented (a greater police presence and regulations on alcohol) to raise awareness more effectively.

Between 2011 and 2013, researchers [24], [26] tested the introduction of stickers in public transport in Kenya (Matutu) backed up by radio campaigns to encourage passengers to monitor drivers’ driving. Four types of sticker were stuck inside vehicles. The messages were very clear: “Don’t let a reckless driver get away”, two in English and two in Kiswahili (the local language). Two local radio stations broadcast awareness raising messages five times per week over the first six months of 2012. The authors estimated that the number of road accidents and victims went down without a precise measure supporting their estimation. They demonstrated an improvement in road safety through regular airing of a memorable and actionable message.

In Zambia [25], a preventive programme targeted child pedestrians in Lusaka, by raising their awareness of the road environment and of the behaviours to adopt to avoid road accidents. Contrary to the interventions in Ghana and Kenya, prevention was based on several different channels of communication. Namely programmes or publicity about road safety on the radio and television. The programmes also proposed concerts and theatre sketches followed by debates with the population.

#### 2 –Training

Three references related to training sessions. Two of them described a single intervention conducted on motorcyclists in Nigeria, at Uyo [27], [28]. The goal of this intervention was to conduct training sessions to learn about road safety signage. These were group sessions and given in the local language. The intervention consisted of readings and exchanges on the identification of road safety signage, as well as how to behave on the roads. A single session was organised in September 2008, two groups were monitored, one (n = 100) took part in the training and the other did not (n = 100). After the intervention, the authors noted that the degree of road safety knowledge rose from 21% to 82% in the group that had taken part in the training course whereas the score went from 19% to 21% in the control group. With regard to the question on behaviours, the study reported that the number of people who drove in a state of fatigue fell from 69% to 42% in the intervention group whereas it rose from 74 to 79% in the control group. With regard to compliance with speed limits, the proportion of people who stated they respected them went from 37.5% to 56.6% in the intervention group but saw no change in the control group (37.5%).

In Rwanda, in 2011, a training course was set up with the purpose of improving coverage of first aid [29]. These courses were designed to demonstrate the ATLS (Advanced Trauma Life Support) method. Training was given to 24 trauma surgeons and 15 trauma nurses. There were two three-day training sessions over two periods (October and November 2015). According to the hospital registry, the main results showed a drop in the number of deaths among the most serious cases following the training sessions.

Other interventions described in the part dedicated to policy and programme safety used training sessions in addition to another intervention [30–32]

#### 3 –Technologies and databases

Two publications dealt with interventions using new technologies to analyse road traffic accidents and improve road safety. These two studies concerned Nigeria.

The first one [33] proposed the development of a web application to monitor road traffic accidents in order to replace hard copy. The goals of this intervention were to provide road safety agents with a monitoring system which would enable them to act faster in the event of an accident (to improve first aid responses) with a system for investigating and recording the events. This system would also enable all the actors to consult the road data and take decisions which would reduce accidents. The necessary variables for monitoring were identified and collected with the federal road safety commission. The data concerning the date of the accident, information on the road and vehicles involved, environment, type of collision, nature of the material damage and personal injuries, referral hospital, and information on the health of the victim(s) were gathered by the policemen on mobile telephone. The system developed showed that those responsible for road safety, and all the other stakeholders, could register, sign in, submit reports and carry out searches on information that had already been entered into the system. Likewise, decision-makers could make requests to carry out developments and reduce accidents. However, the article did not give precise details about the extent of the system’s implantation nor its geographical deployment.

The second study [34] set out an intervention which used Geographic Information Systems (GIS) to gain a better understanding of the scale of road accidents in Nigeria and the variations between states. The objective was to supply elements of decision support. This analysis focused on road traffic accident data between 1990 and 2012, drawing up a ranking between the States of Nigeria. The analysis showed that a better understanding of accident sites had an impact on the total number of accidents, which could be explained by an improvement in traffic conditions, and the success of education and campaigns targeting road safety. The authors noted, however, that injuries were increasingly severe. GISs made it possible to improve knowledge of accidents, which was used to target places as well as awareness raising campaigns. The authors made it clear that they did not know if these analyses were used in the decision-making.

A single article presented an intervention in a real-life situation with the implementation of a data collection system using smartphones in Nigeria [33]. The second article [34], and one of the actions described in the article by Terje [20], presenting complex intersecting interventions, provided assessment methods of accident situations and recommended they be integrated into public policies for road safety. They demonstrated the relevance thereof in their accident analysis and their interest in identifying the targets of preventive action. The article by Idowu and Williams [33], presented an intervention whose objective was to create an electronic monitoring system so that the input of data relating to accidents could replace hard copy. Another advantage was to enable searchable databases to be built to assist in creating actions to be undertaken to reduce accidents. The system developed was based on the use of smartphones by police officers who had an app to enter information about the accident. The variables on the date, time, description of the vehicles involved [34], cause of the accident, environment, type of collision, nature of the injury and lastly the hospital receiving the patient were collected. All this was then sent to a server available for consultation via a web interface authorising access to the database. The system made it possible to account for the number of accidents and victims as well as the severity of the injuries. It demonstrated how important monitoring systems were in reducing road traffic accidents.

#### 4 –Safety policy and programme

Articles with regard to implementing interventions in the legislative field did not form part of our scoping review. The selection dealt mainly with public policies in which laws were passed and the impact analysed. These were only general analyses on several countries, in Africa, and rarely on precise cases. The Anderson et al. article [22], for example, dealt with the effectiveness of public policies in reducing the harm linked to alcohol. Apart from educational and awareness raising measures, one of the major legislative actions was the implementation of a legal limit of alcohol in the blood together with penalties in the event of being over the limit. The effectiveness of this would seem to have been proven but in general terms, on a global scale, without analysing the findings on the scale of a specific country or the African continent. In grey literature [20] there are records of regulations that were implemented in several African countries, such as the legal alcohol limit when driving, speed limits or wearing a seat belt. These were not really interventions as such, rather an analysis of the effectiveness of these regulations in 1998. The authors concluded that in Kenya, Benin, Tanzania, Côte d’Ivoire and Zimbabwe, these laws were but rarely enforced. In the article by Rose-Innes [35] in 1974, an epidemiological analysis was conducted in South Africa on the incidence and severity of head injuries in the framework of speed restrictions and petrol consumption imposed in the context of the oil shock in the 70s. The intervention consisted in reducing fuel consumption by denying access to petrol stations between 6.00 pm and 6.00 am, by limiting fuel reserves to 10 litres per vehicle owner and by reducing speeds from 80km/h to 50 km/h in urban areas and from 112 km/h to 80 km/h in other areas. The effectiveness was visible on fatalities, multiple head injuries and on the behaviour of drivers.

The article by Abegaz et al [36] assessed whether there was an effective reduction in road traffic accidents, morbidity and mortality following the new regulations on road safety introduced in September 2007 in Ethiopia. The regulations included in particular the ban on using a mobile phone, the obligation to use a helmet, to use a seat belt and stepped up enforcement of excessive speeding, drunk driving and carrying dangerous loads. The study showed a statistically significant reduction in accidents and deaths after the regulations were implemented. However, the reduction was often insufficient and implementation of the regulations should be accompanied, according to the authors, by campaigns to raise the public’s awareness and by collaboration between the different sectors.

The article by Bishai [37] presented an intervention on traffic enforcement in Kampala, Uganda. The intervention consisted in the police reinforcing traffic control in four major areas of the capital. Traffic control targeted speed, using measures such as speed cameras, checking dangerous driving, overloaded vehicles and drivers’ possession of a driving licence. The assessment showed that the measures were effective on mortality with an estimated 17% drop in incidence. The authors also referred to the cost-effectiveness of the intervention and asserted that it was the least costly and the most effective compared to other road safety interventions in LMICs.

#### 5 –Highway equipment and improvements

Three articles dealt with the distribution of safety equipment in Tanzania and the world [30], [38], [39]. Three others focused on the installation of highway equipment with the purpose of lowering traffic speed or protecting pedestrians in Uganda, South Africa and Nigeria [31], [40] [32].

In 2008, Liu et al [39] conducted a review on interventions concerning helmet utilization by motorcyclists to reduce mortality and head injuries. They identified 61 studies, most of which were based in developed countries. The article by Sumner et al. [38] described the advantage in drivers’ using equipment that made them easier to be seen by others and other vehicles. Another intervention consisted in introducing protective measures to improve visibility: handing out high-visibility or fluorescent jackets, white helmets, or using dipped-beam headlights throughout the day. Other users received a short five-minute training session on road safety as well.

Another intervention in rural Tanzania organised the distribution of road safety equipment to different categories of the population [30]. Before and after the intervention, the investigators inquired into households situated 200 metres from a main road in the north of Tanzania, in the district of Bagamoyo. 100 motorcyclists were selected to receive high-visibility jackets, two motorcycle helmets and a week’s road safety training. 26 received “back support” for their motorcycle. 2,150 schoolchildren were given reflective bags and 56 teachers were provided with road safety training. Results showed that in the event of an accident the proportion of motorcyclists wearing a helmet had not changed after the intervention (66% before and 63% after) in the area where it had occurred, whereas it had risen in the control area (50% before and 63% after). Broadly speaking, the results did not show a substantial impact, with the authors concluding their article by affirming that despite there being no literature attesting to the effectiveness of education on road safety on the reduction in the number of injuries, it should be included in any general road safety programme.

The article by Obionu in 1985 [32] dealt with highway developments and reports on the erection of bridges to reduce the number of pedestrian accidents. A development of the kind was carried out in Nigeria, in the urban centre of Enugu near a school of 2,500 pupils. A one-week road safety course completed the intervention process. Mutto et al. [40] also studied the impact of a bridge built at Nakawa in 1999. The authors identified several issues linked to insufficient pre-analysis in the development of an overpass for pedestrians. Yet the results showed a significant reduction in the number of people not using the overpass above the nearby motorway.

Lastly, a final study analysed the calming measures installed since 2011 in Durban, South Africa [31]. These developments consisted essentially of speed bumps as well as improvements to the highway. In parallel, road safety training courses were introduced in school curricula as well as employing people to help pedestrians cross the road. After the intervention, it would appear that speed bumps improved safety in the two sectors of the town where the intervention took place. Serious collisions between pedestrians and vehicles dropped by 23% and 22%, whereas fatal collisions fell by 68% and 50% in the districts of Chatsworth and KwaMashu. The median annual rate of accidents per kilometre of road per year decreased from 1.41 to 0.96 and from 2.35 to 1.40 at Chatsworth and at KwaMashu. There was a reduction of 1.6% in the median number of fatal or serious accidents after implementation at Chatsworth whereas at KwaMashu, although the number of collisions fell, the median number rose by 9%.

## Discussion

Our scoping review on the description of interventions to reduce the consequences of road accidents in Africa confirms the low number of interventions on the continent. It was also noted that the implementation of interventions is relatively recent as 13 of the 23 articles have been published since 2011. They correspond in part to the impact of the Decade of Action for Road Safety (2011–2020) and focus on the five pillars. This scoping review is a significant complement to the systematic review published in 2016 by Adeloye. The latter retained only three interventions in Africa out of the 18 chosen for their publication dedicated to LMICs. The same is true regarding the low number of interventions, despite numerous calls for papers published in scientific journals [41–43], and it is noticeable that little intervention research is carried out in Africa, with the exception of that funded by the Bloomberg Philanthropies Foundation, which only concerns a few countries. Since 2007, in fact, the Foundation has developed interventions in seven countries around the world, two of which are in Africa (Ghana and Ethiopia). Bloomberg Philanthropies’ goals are to work with local partners to implement road safety activities which respect the mechanisms of high quality assessments to measure their impact. The programmes focus on five fields of intervention: behaviour (helmet use, seat belt use, alcohol consumption and speed), infrastructures, sustainable urban transport, vehicle standards and policy strengthening. It is in the latter field that interventions were conducted in Ghana and Ethiopia by reactivating lighting on the main roads to Accra and by establishing an inter-agency Road Safety Council chaired by the Deputy Mayor in Addis Ababa. [44]

The geographic analysis of the interventions identified in this scoping review revealed a concentration of intervention research in Anglophone Africa supported in part by the Bloomberg Foundation and the Road Safety Programme. Francophone Africa, in the west or centre of the continent, presented very few interventions. Besides Benin, Francophone Africa was not represented despite mortality rates that were amongst the highest in the last WHO assessment [2]. Could it be that the distribution of aid and the mobilisation of intervention research are unfair in that they do not necessarily target the countries with the greatest needs [45]? One of the explanations for this inequality of treatment might be that countries in this part of the continent are not eligible for calls for projects from the Bloomberg Foundation and the Road Safety Programme. But also because most of these countries, if they try to apply the WHO recommendations, do not have reliable and regular databases on the number of accidents, characteristics of the victims or monitoring of their health. In this context, it is impossible to assess the effectiveness of an intervention on the number of accident victims, apart from integrating a monitoring system before the intervention to assess the impact. Similarly, assessing the cost-effectiveness of an intervention can be flawed, as Bishai et al mention in their study [37]. Integrating a monitoring system with an intervention in a preventive field is possible; several articles demonstrated this in this scoping review and in other papers [46] but this supposes a greater implementation time, and would therefore be more costly, as well as working in close collaboration with the police, rescue and health authorities of the country. In this respect, it would be legitimate to recommend that the first interventions to conduct in the framework of the Decade of Action for Road Safety and SDGs would be to develop a system of reliable data collection, specifically adapted to the context of each country not currently possessing an efficient system. WHO and the International Automobile Federation stated in a recent report that “*Without accurate data, it is impossible to develop effective road safety interventions, and it is impossible to evaluate the effectiveness of those interventions that are in place. In most sub-Saharan African countries, researchers cannot rely on the data available from government sources to accurately quantify the problem, measure the impact of injury prevention strategies, or appreciate how the patterns of injury evolve over time.”[47]*

This scoping review also reveals that several interventions perform combined activities [22],[20], illustrating that many complex leverages of action are in play. It is more difficult to assess a combination of interventions, however, and to reproduce them in other contexts as a result. Our analyses of the articles reveal that the interventions carried out are rather lacking in detailed description and endeavour rather to present the objectives and framework in which they were carried out. The intervention fidelity is therefore impossible to understand. Apart from the article by Bishai et al [37], or those by Habyarimana [26], few articles mention the details of the intervention’s implementation, its possible reproducibility and the role of the contextual aspects. Fewer than five articles presented a detailed context as provided by Ridde et al [13]. The interventions were therefore described too simply, minimally and with no details relative to the context, which is essential for scaling up or replication. We know, in fact, that there are numerous issues of transferability in intervention research [48].

Considering the assessment methods, the greatest proportion provided meta-analyses from which it was difficult to extract the initial data and therefore to reproduce in another context. In the end, nine articles focused on effectiveness. The authors tended to validate the outcomes of their interventions without necessarily supporting them by robust assessments which were themselves difficult to carry out, given that there was no monitoring of accident and victim numbers, or resulted from an estimation. We made the choice of including them in this scoping review, making it clear in the synthetic table the type of assessment and demonstration of their effectiveness or otherwise. The existence of these interventions and their implementation remain relevant, useful and rare in Africa. These experiments will serve to build other intervention projects which should be adapted to the countries’ contexts.

All the fields of prevention and types of intervention had something to do with awareness raising, in particular via the media or communication. However, in the examples used in this scoping review, it can be noted that the media or communication tools involved were adapted to the contexts of the countries in which they were applied, like the stickers in Kenya, or raising awareness via community radio stations in Zambia. If these actions appeared effective, they remain difficult to assess and generalise. The second field which seems to be the most effective relates to basic first aid training for nurses and emergency doctors when treating casualties. Lastly, the third field is traffic enforcement with a dissuasive effect through the presence of security forces and checks. This combination of interventions is reminiscent of Haddon’s conceptual framework [49] where, although it was developed in the framework of the transport sector, corresponds to a public health prevention approach [50]. Prevention is apparent both in the potential to prevent accidents (pre-crash phase), a secondary prevention corresponding to the potential to attenuate the impact of the accident in the event of a collision and tertiary prevention corresponding to the potential to save lives.

## Conclusion

The success of interventions on traffic enforcement must be associated with the context of the countries, given that differences exist in the effectiveness of the administrative and legal systems within countries on the African continent. Populations’ level of resources is also crucial in the use of fines. The impact would be negligible, would indeed worsen living conditions, if applied to the most disadvantaged populations. Lastly, road safety education as well as raising traffic protection awareness seem to be the interventions which are most adapted to African countries even if a strategy for changing behaviours must be adopted, appropriate to each socio-economic and national cultural context. The overall analysis of this scoping review reveals that there is a lack of interventions in the field of road safety on the continent, even though the World Decade for Road Safety and many awareness raising activities by the scientific communities had stressed the urgency of the situation. Available funding focusing specifically on the subject is certainly insufficient to produce new knowledge emanating from research. A final point to take into account is that the few interventions conducted could be better assessed so that elements of replication could be obtained in order for the experiments to be directed to other places and on other scales.

## Supporting information

S1 Appendix Research strategy. (PDF)

S2 Appendix Prisma checklist. (PDF)

S3 Appendix TIDieR checklist. (XLSX)
