# Supplementary material for: What interventions are required to reduce road traffic injuries in Africa? A scoping review of the literature
Source: PLoS One. 2018 Nov 30;13(11):e0208195. doi: 10.1371/journal.pone.0208195 (PMC6267971; doi:10.1371/journal.pone.0208195)
Supplement: S1 Appendix — (PDF) [file pone.0208195.s001.pdf]

Appendix 1

Database	Research strategy
**Science Direct**	("accident traffic" OR "road traffic injury" OR "Road safety" OR "Road crash" OR "road accident") AND ("accident prevention" OR "accessibility of health services" OR "access to health care" OR "accessibility, health services" OR "health intervention" OR "development, policy" OR "efficiency, program" OR "effectiveness research, comparative" OR "effectiveness" OR "efficiency" OR "evidence-based") AND<br>("africa" OR "west africa" OR "Burundi" OR "republic of Burundi" OR "Angola" OR "Algeria" OR "Benin" OR "republic of Benin" OR "Comoros" OR "iles Comores" OR "Cameroon" OR "republic of Cameron" OR "united republic of Cameroon" OR "Egypt" OR "arab republic of Egypt" OR "Burkina Faso" OR "burkina faso" OR "Djibouti" OR "republic of Djibouti" OR "Central African Republic" OR "Libya" OR "Cape Verde" OR "republic of cape verde" OR "Eritrea" OR "Chad" OR "Morocco" OR "Cote d'Ivoire" OR "ivory coast" OR "Ethiopia" OR "federal democratic republic of Ethiopia" OR "Congo" OR "congo Brazzaville" OR "congo Kinshasa" OR "Sudan" OR "republic of the sudan" OR "Gambia" OR "republic of the gambia" OR "Kenya" OR "republic of Kenya" OR "Democratic Republic of the Congo" OR "Tunisia" OR "Ghana" OR "republic of Ghana" OR "Madagascar" OR "Guinea" OR "Malawi" OR "republic of Malawi" OR "Equatorial Guinea" OR "republic of equatorial guinea" OR "Guinea-Bissau" OR "republic of guinea Bissau" OR "Mauritius" OR "Gabon" OR "Liberia" OR "republic of Liberia" OR "Mozambique" OR "republic of Mozambique" OR "Sao Tome and Principe" OR "Mali" OR "Reunion" OR "Botswana" OR "Mauritania" OR "Rwanda" OR "republic of Rwanda" OR "Lesotho" OR "kingdom of Lesotho" OR "Niger" OR "republic of niger" OR "Seychelles" OR "Namibia" OR "republic of Namibia" OR "Nigeria" OR "federal republic of Nigeria" OR "Somalia" OR "St Helena" OR "saint Helena" OR "Uganda" OR "republic of Uganda" OR "Swaziland" OR "Senegal" OR "republic of Senegal" OR "United Republic of Tanzania" OR "united republic of Tanzania" OR "Sierra Leone" OR "republic of sierra leone" OR "Zambia" OR "republic of Zambia" OR "Togo" OR "Zimbabwe" OR "republic of Zimbabwe" OR "zimbabwe rhodesia").
**PubMed**	((((((accident, traffic[MeSH Terms]) OR road traffic injury[Title/Abstract]) OR road crash[Title/Abstract]) OR road safety) OR road accident))<br>AND ((((((((((accident prevention[MeSH Terms]) OR accessibility of health services[MeSH Terms]) OR access to health care[MeSH Terms]) OR accessibility, health services[MeSH Terms]) OR health intervention[Title/Abstract]) OR development, policy[MeSH Terms]) OR efficiency, program[MeSH Terms]) OR effectiveness research, comparative[MeSH Terms]) OR effectiveness[Title/Abstract]) OR efficiency[MeSH Terms])) AND<br>(((africa[MeSH Terms]) OR africa, sub saharan[MeSH Terms]) OR low income population[MeSH Terms]) OR class population, middle[MeSH Terms]) OR countries, developing[MeSH Terms]) OR countries, less developed[MeSH Terms]) OR countries, under developed[MeSH Terms]) OR less developed countries[MeSH Terms]) OR developing countries[MeSH Terms]) OR countries, third world[MeSH Terms]) OR africa, western[MeSH Terms]) OR burundi[MeSH Terms]) OR republic of burundi[MeSH Terms]) OR angola[MeSH Terms]) OR benin[MeSH Terms]) OR republic of benin[MeSH Terms]) OR comoros[MeSH Terms]) OR cameroon[MeSH Terms]) OR republic of cameron[MeSH Terms]) OR united republic of cameroon[MeSH Terms]) OR egypt[MeSH Terms]) OR arab republic of egypt[MeSH Terms]) OR burkina faso[MeSH Terms]) OR burkina fasso[MeSH Terms]) OR djibouti[MeSH Terms]) OR republic of djibouti[MeSH Terms]) OR central african republic[MeSH Terms]) OR libya[MeSH Terms]) OR cape verde[MeSH Terms]) OR republic of cape verde[MeSH Terms]) OR eritrea[MeSH Terms]) OR chad[MeSH Terms]) OR morocco[MeSH Terms]) OR cote d ivoire[MeSH Terms]) OR cote d'ivoire[MeSH Terms]) OR ivory coast[MeSH Terms]) OR ethiopia[MeSH Terms]) OR federal democratic republic of ethiopia[MeSH Terms]) OR congo brazzaville[MeSH Terms]) OR congo[Title/Abstract]) OR congo kinshasa[MeSH Terms]) OR republic of the sudan[MeSH Terms]) OR sudan[MeSH Terms]) OR gambia[MeSH Terms]) OR republic of the gambia[MeSH Terms]) OR kenya[MeSH Terms]) OR republic of kenya[MeSH Terms]) OR democratic republic of the congo[MeSH Terms]) OR tunisia[MeSH Terms]) OR ghana[MeSH Terms]) OR republic of ghana[MeSH Terms]) OR madagascar[MeSH Terms]) OR guinea[MeSH Terms]) OR malawi[MeSH Terms]) OR republic of malawi[MeSH Terms]) OR equatorial guinea[MeSH Terms]) OR republic of equatorial guinea[MeSH Terms]) OR guinea bissau[MeSH Terms]) OR guinea bissau,

	republic of[MeSH Terms]) OR mauritania[MeSH Terms]) OR mauritius[MeSH Terms]) OR gabon[MeSH Terms]) OR gabonese republic[MeSH Terms]) OR republic of south africa[MeSH Terms]) OR south africa[MeSH Terms]) OR liberia[MeSH Terms]) OR republic of liberia[MeSH Terms]) OR mozambique[MeSH Terms]) OR republic of mozambique[MeSH Terms]) OR (sao tome and principe[MeSH Terms])) OR mali[Title/Abstract]) OR reunion[MeSH Terms]) OR bostwana[Title/Abstract]) OR republic of rwanda[MeSH Terms]) OR rwanda[MeSH Terms]) OR kingdom of lesotho[MeSH Terms]) OR lesotho[MeSH Terms]) OR niger[MeSH Terms]) AND seychelles[MeSH Terms]) OR republic of namibia[MeSH Terms]) OR namibia[MeSH Terms]) OR federal republic of nigeria[MeSH Terms]) OR nigeria[MeSH Terms]) OR somalia[MeSH Terms]) OR saint helena[MeSH Terms]) OR republic of uganda[MeSH Terms]) OR uganda[MeSH Terms]) OR swaziland[MeSH Terms]) OR republic of senegal[MeSH Terms]) OR senegal[MeSH Terms]) OR tanzania[MeSH Terms]) OR united republic of tanzania[MeSH Terms]) OR republic of sierra leone[MeSH Terms]) OR sierra leone[MeSH Terms]) OR republic of zambia[MeSH Terms]) OR zambia[MeSH Terms]) OR togo[MeSH Terms]) OR togolese republic[MeSH Terms]) OR republic of zimbabwe[MeSH Terms]) OR zimbabwe[MeSH Terms]) OR zimbabwe rhodesia[MeSH Terms])
**EMBASE**	(accident traffic OR road traffic injury OR road accident OR road safety) AND (health intervention OR health polic* OR health program* OR health prevention) AND ("africa" OR "west africa" OR "Burundi" OR "republic of Burundi" OR "Angola" OR "Algeria" OR "Benin" OR "republic of Benin" OR "Comoros" OR "iles Comores" OR "Cameroon" OR "republic of Cameron" OR "united republic of Cameroon" OR "Egypt" OR "arab republic of Egypt" OR "Burkina Faso" OR "burkina faso" OR "Djibouti" OR "republic of Djibouti" OR "Central African Republic" OR "Libya" OR "Cape Verde" OR "republic of cape verde" OR "Eritrea" OR "Chad" OR "Morocco" OR "Cote d'Ivoire" OR "ivory coast" OR "Ethiopia" OR "federal democratic republic of Ethiopia" OR "Congo" OR "congo Brazzaville" OR "congo Kinshasa" OR "Sudan" OR "republic of the sudan" OR "Gambia" OR "republic of the gambia" OR "Kenya" OR "republic of Kenya" OR "Democratic Republic of the Congo" OR "Tunisia" OR "Ghana" OR "republic of Ghana" OR "Madagascar" OR "Guinea" OR "Malawi" OR "republic of Malawi" OR "Equatorial Guinea" OR "republic of equatorial guinea" OR "Guinea-Bissau" OR "republic of guinea Bissau" OR "Mauritius" OR "Gabon" OR "Liberia" OR "republic of Liberia" OR "Mozambique" OR "republic of Mozambique" OR "Sao Tome and Principe" OR "Mali" OR "Reunion" OR "Botswana" OR "Mauritania" OR "Rwanda" OR "republic of Rwanda" OR "Lesotho" OR "kingdom of Lesotho" OR "Niger" OR "republic of niger" OR "Seychelles" OR "Namibia" OR "republic of Namibia" OR "Nigeria" OR "federal republic of Nigeria" OR "Somalia" OR "St Helena" OR "saint Helena" OR "Uganda" OR "republic of Uganda" OR "Swaziland" OR "Senegal" OR "republic of Senegal" OR "United Republic of Tanzania" OR "united republic of Tanzania" OR "Sierra Leone" OR "republic of sierra leone" OR "Zambia" OR "republic of Zambia" OR "Togo" OR "Zimbabwe" OR "republic of Zimbabwe" OR "zimbabwe rhodesia")
**French Public health database**	([accident circulation] ; [afrique])
**PsycInfo**	Road traffic injury AND africa
**MedNar**	Road traffic injury africa
**EBSCOhost**	(road traffic injury OR road traffic safety OR road traffic accident OR accident tr) AND (health intervention OR health polic* OR health program* OR health prevention) AND (africa OR west africa OR low income OR middle income OR developing countries)<br>Mode de recherche : Boléen/phrase
